# Association between Oral Health Status and Relative Handgrip Strength in 11,337 Korean

**DOI:** 10.3390/jcm10225425

**Published:** 2021-11-20

**Authors:** Ji-Eun Kim, Na-Yeong Kim, Choong-Ho Choi, Ki-Ho Chung

**Affiliations:** 1 Department of Preventive and Public Health Dentistry, Chonnam National University School of Dentistry, Gwangju 61186, Korea; angel761@chonnam.ac.kr (J.-E.K.); 216622@chonnam.ac.kr (N.-Y.K.); hochoi@chonnam.ac.kr (C.-H.C.); 2 Dental Science Research Institute, Chonnam National University, Gwangju 61186, Korea

**Keywords:** KNHANES, periodontitis, present teeth, relative handgrip strength

## Abstract

Grip strength is a simple indicator of physical strength and is closely associated with systemic health. Conversely, oral health has also been reported to have an important association with systemic health. The present study aimed to assess the effect of oral health status on relative handgrip strength. The data pertaining to 11,337 participants were obtained by means of the seventh Korea National Health and Nutrition Survey (2016 to 2018). Oral health status was evaluated on the basis of the presence of periodontitis and number of remaining teeth (PT, present teeth). Relative handgrip strength was evaluated by means of a digital dynamometer and the value pertaining to the lower 25% of measurements was used as the quartile by gender. The association between oral health status and relative handgrip strength was evaluated by means of multiple regression analysis and multiple logistic regression analysis with covariate correction. Analysis of the crude model revealed a significant association in the group of patients with periodontal disease (odds ratio = 1.69, 95% confidence interval: 1.51–1.89). However, analysis with adjusted covariates revealed that the association was not statistically significant. Moreover, statistical analysis after adjustment for covariates revealed a consistent correlation between PT and relative handgrip strength as categorical and continuous variables. Hence, the present study observed a significant association between oral health status and relative handgrip strength among the Korean adult population.

## 1. Introduction

Advancements in the field of science have led to an increase in human lifespan, and emphasis on the quality of life is important for the pursuit of a healthy life. Correspondingly, physical function plays an important role in the quality of life [1] and handgrip strength is widely used to conveniently evaluate physical functions [2,3]. Handgrip strength is divided into two categories: absolute handgrip strength and relative handgrip strength. The latter is computed by dividing absolute handgrip strength by the individual’s body mass index (BMI). Relative handgrip strength has been recommended to address the disturbance in muscle strength attributable to body mass as well as the health risks associated with weight gain and muscle weakness [4,5]. Moreover, recent studies have demonstrated the association between relative handgrip strength and systemic diseases. A study by Lawman et al. [6] reported the association between handgrip strength and biomarkers of cardiovascular disease using the data obtained from the National Health and Nutrition Examination Survey (2011–2012). Another study by Yi et al. [7] reported the association between handgrip strength and metabolic syndrome using the data obtained from the Korea National Health and Nutrition Survey (KNHANES) (2014–2015). Furthermore, a prospective cohort study of Japanese subjects by Manda et al. [8] reported that handgrip strength could be used to predict the incidence of prediabetes.

Similarly, oral health is closely related not only to an individual’s physical fitness [9], but also to systemic diseases. Periodontal disease, one of the most common oral diseases, is a chronic inflammatory disease that can affect humans throughout life [10]. A previous study by Falcao and Bullón [11] observed that the effects of periodontal disease were not limited to the oral cavity alone. The aforementioned study observed associations between periodontal disease and several systemic health conditions and diseases, including cardiovascular disease, diabetes, rheumatoid arthritis, and respiratory disease. Previous studies have reported that the overall nutritional status and quality of life improved with the improvement in chewing ability, which represents the oral health status [12]. In addition, other studies have reported that comfortable chewing warrants the functionality of more than 20 remaining teeth (PT, present teeth) [13]. Moreover, the association between dental occlusion and physical fitness has been reported by Yamaga et al. [14], and another study by Kamdem et al. [15] reported that PT and masticatory function were associated with diabetes. In accordance with the results reported by the aforementioned studies, oral health is very important and periodontal disease and PT are representative indicators of the oral health status [16,17].

In literature, only a limited number of studies have reported the association between handgrip strength and oral health status. The relationship between absolute handgrip strength and PT in the Korean population was reported by Shin [18]. Furthermore, the same association was reported by a similar study with a smaller sample size [16] and another study that performed the statistical analysis after adjustment for a few covariates [19]. However, few studies have confirmed the association between periodontal disease and PT (indicators of oral health status) and the relative handgrip strength (indicator of healthy functioning of the body).

Hence, the purpose of the current study was to assess the association between oral health and relative handgrip strength in adults ≥19 years of age. To the best of our knowledge, this is the first domestic study that analyzed the relationship between periodontal disease and PT and relative handgrip strength after adjusting for various covariates in a representative sample of the general population.

## 2. Materials and Methods

### 2.1. Study Population

**Figure 1:**
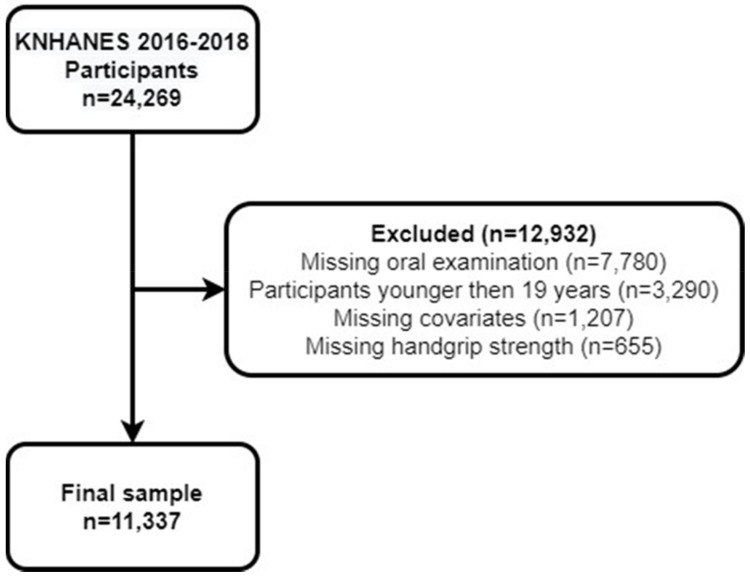
Flow chart of the selection process.

The current study obtained data pertaining to the time period from 2016 to 2018 by means of the seventh KNHANES. Among a total of 24,269 prospective subjects (8150, 8127, and 7992 participants pertaining to the years 2016, 2018, and 2018, respectively), the present study excluded 12,932 subjects who did not undergo oral and handgrip strength evaluations or had missing covariates. The final sample included 11,337 adult participants ≥19 years of age (Figure 1).

The current study was approved by the Research Ethics Review Committee of the Korea Centers for Disease Control and Prevention (2018-01-03-P-A). Written informed consent was obtained from all the subjects prior to the survey.

### 2.2. Handgrip Strength

Handgrip strength was evaluated by means of a digital dynamometer (Digital grip strength dynamometer, T.K.K 5401, Takei Kikai Kogyo Co., Ltd., Tokyo, Japan). In accordance with the guidelines for evaluation [20], the measurement was performed in a standing position and both hands were crossed three times.

For evaluation, the current study employed relative handgrip strength that was computed by dividing the respective maximum handgrip strength of the dominant hand by the corresponding BMI and expressed as kg_BMI_.

The reference value used for the categorization of low relative handgrip strength was based on the results reported by previous research [21]. The value pertaining to the lower 25% of measurements was used as the quartile by gender.

### 2.3. Oral Examination

The oral examinations were performed, and results were documented by a trained dentist, according to the guidelines of KNHANES [22]. Periodontitis was diagnosed using the Community Periodontal Index (CPI) [23], which was determined using the WHO CPI probe. The scores were marked as follows: 0: healthy periodontal tissue; 1: periodontal tissue with bleeding on probing; 2: periodontal tissue with calculus formation; 3: periodontal tissue with pocket depth (PD) of 4.0–5.0 mm; and 4: periodontal tissue with PD > 5 mm. It was divided into two categories: Yes, for codes ≥3 (codes 3 and 4) and no, for codes below 3 (codes 0, 1, and 2). The PT was calculated by adding up all the remaining teeth (maximum of 28 teeth), excluding the third molars. Subsequently, the subjects were classified into three groups on the basis of the same: 0–9 teeth, 10–19 teeth, and 20–28 teeth.

### 2.4. Covariates

The general characteristics pertaining to the subjects included gender, age, educational level, and household income. The variables concerning general health behaviors included smoking (nonsmoker, former smoker, current smoker), alcohol consumption (nondrinker, alcohol consumption once per month, alcohol consumption ≥ twice per month), exercise (moderate-intensity physical activity for a minimum duration of 2 h 30 min, or high-intensity physical activity for a minimum duration of 1 h 15 min, or a combination of moderate- and high-intensity physical activity per week), BMI (weight/height^2^), and presence of comorbidities (number of diagnosed cases of chronic diseases such as hypertension, diabetes, stroke, myocardial infarction or angina pectoris, arthritis, and cancer). Variables pertaining to oral health behaviors included the frequency of brushing teeth per day, use of oral hygiene products (use of dental floss, interdental toothbrush, mouth rinse, electric toothbrush, and other products), chewing problems, speaking problems, dental visits during the past year, and self-perceived oral health status.

### 2.5. Statistical Analyses

KNHANES is a complex sample survey and data analysis was performed in consideration of the stratification variable, cluster variable, and weight, owing to the complex sample design. The subjects were categorized on the basis of their respective relative handgrip strength. The sociodemographic characteristics of the participants were analyzed using the *t*-test or chi-square test. The present study employed multiple regression analysis to confirm the association between relative handgrip strength as a continuous variable and PT as a continuous and categorical variable. Moreover, the association between lower relative handgrip strength and PT or categorical periodontal disease as continuous and categorical variables among the subjects with handgrip strength below the lower quartile was assessed using multiple logistic regression analysis. Adjustment for the variables pertaining to general characteristics, general health behaviors, and oral health behaviors was performed to determine the odds ratio (OR) of oral health status and relative handgrip strength. The statistical significance was set at *p* < 0.05. In the present study, SAS 9.4 program (SAS Institute, Cary, NC, USA) was used to perform the statistical analysis.

## 3. Results

### 3.1. General Characteristics of the Participants

The general characteristics of the subjects are presented in Table 1. The present study involved a total of 11,337 participants with an average age of 49.81 ± 0.31 years. The group with low relative handgrip strength, which included the subjects with handgrip strength below the lower quartile, had greater age, lower educational levels, lower household income, no exercise routine, obesity, greater number of comorbidities, fewer PT, and a higher proportion of patients with periodontal disease, compared to the group with high relative handgrip strength, which included the subjects with handgrip strength above the lower quartile (top 75%).

### 3.2. Association between PT and Relative Handgrip Strength as a Continuous Variable

The effects of PT as a continuous and categorical variable on relative handgrip strength as a continuous variable are shown in Table 2. Analysis of the crude model with PT as a continuous variable revealed that increase in PT by one tooth effected significant corresponding increase in the relative handgrip strength by 0.014 kg_BMI_ (*p* < 0.0001). Furthermore, analysis of the model adjusted for covariates revealed that despite the decrease in regression coefficient, increase in PT by one tooth effected significant corresponding increase in the relative handgrip strength by 0.003 kg_BMI_ (*p* < 0.0001).

Regarding the effects of PT as a categorical variable, analysis of the crude model revealed significantly lower relative handgrip strength in the group with fewer teeth than in the group with 20–28 teeth (10–19: −0.179, *p* < 0.0001; 0–9: −0.183, *p* < 0.0001). Moreover, analysis of the model adjusted for covariates revealed a negative correlation (10–19: −0.038, *p* = 0.0006; 0–9: −0.040, *p* = 0.0056).

### 3.3. Association between Oral Health Status and Low Relative Handgrip Strength among the Subjects with Handgrip Strength Below the Lower Quartile (Lower 25%)

The results of multivariate logistic regression analysis of the effects of oral health status on the risk of low relative handgrip strength among the subjects with handgrip strength below the lower quartile (lower 25%) are shown in Table 3. Analysis of the crude model revealed a significant association between periodontal disease and the risk of lower relative handgrip strength (OR = 1.69, 95% confidence interval [CI]: 1.51–1.89). However, analysis of the model adjusted for covariates did not reveal the same association.

Analysis of the crude model with PT as a continuous variable revealed that increase in PT by one tooth effected a corresponding decrease in the OR regarding the risk of low handgrip strength by 0.91 times. Moreover, analysis of the model adjusted for covariates revealed an association between the variables with an OR of 0.97 (CI: 0.96–0.99). Analysis of the model adjusted for covariates with PT as a categorical variable revealed a significant association between PT and the risk of lower relative grip strength among the subjects with PT of 10–19 (OR = 1.34, CI: 1.10–1.62) and 0–9 (OR = 1.29 CI: 1.00–1.67), compared to those with PT of 20–28 (ref).

## 4. Discussion

The current study confirmed the association between oral health status and relative handgrip strength through analysis of national representative data after adjusting for several covariates. Moreover, no previous Korean study has analyzed the association between oral health status, evaluated by means of PT and the presence/absence of periodontal disease, and relative handgrip strength after adjusting for several covariates. The current results indicate a significant association between oral health status and relative handgrip strength in the Korean adult population.

Handgrip strength is a representative measurement item that can be used to evaluate internal strength. In addition, recent studies have reported a correlation between grip strength and mobility, chronic disease morbidity, disability in old age, and total mortality [24,25]. In the current study, handgrip strength was measured in a standing position. This method of evaluation can assess muscle strength of the core and lower body [6]. Hence, the results can reflect the overall body strength. Moreover, disturbance in muscle strength according to the respective body mass could be excluded through the utilization of relative handgrip strength. Several previous studies have employed relative handgrip strength for evaluations. A study by Alley et al. [3] reported that relative handgrip strength was better suited for the evaluation of weakness than absolute handgrip strength. Moreover, a study by Lawman et al. [6] reported that relative handgrip strength is a useful tool that can be employed in the public health evaluation of muscle mass. Accordingly, the present study endeavored to reflect the exact status of physical fitness of the subjects using relative handgrip strength.

The present regression model, adjusted for covariates, revealed a correlation between PT and relative handgrip strength and showed that the effect of PT was continuous and categorical on relative handgrip strength. With regard to the association between PT as a continuous variable and relative handgrip strength, the current results established that relative handgrip strength significantly increased with the corresponding increase in PT in all models. Among the three categories of subjects with PT of 0–9, 10–19, and 20–28, the groups with fewer teeth displayed a tendency to have lower relative handgrip strength than the group with a higher number of teeth. A study by Shin [18] stated that assessment of the relationship between number of teeth and absolute handgrip strength in Korean adults revealed a significant association between greater number of remaining teeth and greater absolute handgrip strength. The aforementioned results imply that the current results are concurrent with the results of previous studies.

Conversely, with reference to the logistic regression model that assessed the relative handgrip strength as a categorical variable and evaluated the effect of periodontal disease on relative handgrip strength, the association between periodontal disease and relative handgrip strength was confirmed through analysis of the crude model. A previous study by Eremenko et al. [19] reported a relationship between clinical adhesion loss and relative handgrip strength and the results were concurrent with the current results. Nonetheless analysis of the model adjusted for covariates did not reveal any significant results. Furthermore, a previous systematic review [11] has reported that periodontal disease is associated with several other factors. Consequently, other covariates pertaining to the subjects might have a greater influence on the results of the present study.

The current study observed an association between PT and relative handgrip strength, regardless of the status of continuous or categorical variable, as PT was a categorical variable in the crude model and the model adjusted for covariates. A study of Chinese individuals below the age of 60 years by Zhou et al. [26] employed the average number of missing teeth as the cut-off value for tooth loss and reported an association between tooth loss and relative handgrip strength. The abovementioned results were similar to the current results. The present study was based on two previous studies: a study by Elias et al. [13], which considered the presence of a minimum of 20 teeth as the criterion for masticatory function, and a study by Peres et al. [27], which considered the presence of 10 or fewer natural teeth as the criterion for masticatory function. Thus, the authors are of the opinion that better reflection of oral health status of the subjects was achieved through the categorization of subjects on the basis of PT into three classes, i.e., 0–9, 10–19, and 20–28. In addition, the current results confirm the association between PT and relative handgrip strength as categorical and continuous variables, respectively [16,18,19,26,28,29]. Analysis of the model adjusted for covariates with PT as a categorical variable revealed that the OR pertaining to the group with PT of 0–9 was slightly lower than that of the group with PT of 10–19. The authors are of the opinion that the current analysis involved subjects with a wide range of age and gender differences, such as menopause-related differences (occurrence or absence) in women, which might have influenced the outcomes, despite the adjustments for age and gender. The current study tried to understand the general trends in relation to oral health and relative handgrip strength in the Korean adult population. The scenario warrants further research to confirm the association between variables by categorizing the subjects on the basis of age and gender.

The putative mechanisms concerning the association between oral health and handgrip strength are diverse and remain ambiguous. Recently, a systematic review reported that oral health status and physical fitness and had a bidirectional relationship [9]. A study by Yamaguchi et al. [30] reported a strong association between tooth loss and thickness of the muscle mass with reference to the main masticatory muscle. Furthermore, a study of Japanese population by Yoshino et al. [31] reported that masticatory force was associated with handgrip strength. Additionally, it has been reported that the masticatory discomfort attributable to tooth loss affects the nutritional status, owing to improper dietary habits [12]. In addition, previous studies have reported that the consumption of branched-chain amino acids is associated with handgrip strength [32]. Hence, the direct or indirect effect of tooth loss on physical strength can explain this association with handgrip strength to a certain extent.

According to an alternative putative mechanism, periodontal disease and handgrip strength share common risk factors, such as systemic inflammatory conditions. A previous study by Visser et al. [33] reported that muscle mass and muscle strength in the elderly were related to the concentrations of interleukin-6 and tumor necrosis factor-α. High levels of the aforementioned inflammatory factors were observed in patients with periodontal disease [11].

The present study has certain limitations. The current study was cross-sectional in nature and a causal relationship between the factors could not be determined. Confirmation of the causal relationship between the two factors warrants a longitudinal study.

Nevertheless, the results of the present study correspond to the entire national population, as the oral health status and relative handgrip strength of 11,337 adults was assessed using large-scale data. Moreover, to the best of our knowledge, this is the first study to confirm the association between relative handgrip strength, which denotes physical strength, and oral health status, which was assessed by way of PT and periodontal disease, through analyses after adjustment for the same covariate.

## 5. Conclusions

The current study verified a significant association between oral health status, evaluated using periodontal disease and PT, and handgrip strength, which represents physical strength, among Korean adults. Promotion and maintenance of oral health is necessary to preserve physical strength, which is essential for a healthy life.

## Figures and Tables

**Table 1:** General characteristics of the subjects.

Variables	Total (*n* = 11,337)	High Relative Handgrip Strength (*n* = 8846)	Low Relative Handgrip Strength (*n* = 2491)	*p*-Value
General characteristics				
Age	49.81 ± 0.31	47.05 ± 0.28	59.67 ± 0.52	<0.0001 ^a^
19–39	3399 (29.98)	3008 (34.00)	391 (15.70)	<0.0001
40–64	5426 (47.86)	4570 (51.66)	856 (34.36)	
≥65	2512 (22.16)	1268 (14.33)	1244 (49.94)	
Educational level				
Primary	2036 (17.96)	1046 (11.82)	990 (39.74)	<0.0001
Middle	1083 (9.55)	778 (8.79)	305 (12.24)	
High	3742 (33.01)	3121 (35.28)	621 (24.93)	
College +	4476 (39.48)	3901 (44.10)	575 (23.08)	
Household income				
Lowest quartile	1964 (17.32)	1125 (12.72)	839 (33.68)	<0.0001
Lower middle quartile	2740 (24.17)	2084 (23.56)	656 (26.33)	
Upper middle quartile	3241 (28.59)	2690 (30.41)	551 (22.12)	
Highest quartile	3392 (29.92)	2947 (33.31)	445 (17.86)	
General health behaviors				
Smoking				
Nonsmoker	6912 (60.97)	5266 (59.53)	1646 (66.08)	<0.0001
Former smoker	2395 (21.13)	1853 (20.95)	542 (21.76)	
Current smoker	2030 (17.91)	1727 (19.52)	303 (12.16)	
Alcohol consumption				
Nondrinker	2979 (26.28)	1946 (22.00)	1033 (41.47)	<0.0001
Once per month	3233 (28.52)	2572 (29.08)	661 (26.54)	
≥Twice per month	5125 (45.21)	4328 (48.93)	797 (32.00)	
Exercise				
No	6377 (56.25)	4720 (53.36)	1657 (66.52)	<0.0001
Yes	4960 (43.75)	4126 (46.64)	834 (33.48)	
BMI (kg/m^2^)				
<18.5	426 (3.76)	399 (4.51)	27 (1.08)	<0.0001
18.5 to <25	6989 (61.65)	5974 (67.53)	1015 (40.75)	
≥25	3922 (34.59)	2473 (27.96)	1449 (58.17)	
Comorbidity				
0	7431 (65.55)	6421 (72.59)	1010 (40.55)	<0.0001
1	2402 (21.19)	1686 (19.06)	716 (28.74)	
≥2	1504 (13.27)	739 (8.35)	765 (30.71)	
Oral health behaviors				
Frequency of brushing teeth per day				
≤1	1086 (9.58)	672 (7.60)	414 (16.62)	<0.0001
2	4365 (38.50)	3321 (37.54)	1044 (41.91)	
≥3	5886 (51.92)	4853 (54.86)	1033 (41.47)	
Use of oral hygiene products				
0	5156 (45.48)	3697 (41.79)	1459 (58.57)	<0.0001
1	3975 (35.06)	3250 (36.74)	725 (29.10)	
≥2	2206 (19.46)	1899 (21.47)	307 (12.32)	
Chewing problem				
Comfortable	8940 (78.86)	7267 (82.15)	1673 (67.16)	<0.0001
Uncomfortable	2397 (21.14)	1579 (17.85)	818 (32.84)	
Speaking problem				
Comfortable	10,533 (92.91)	8405 (95.01)	2128 (85.43)	<0.0001
Uncomfortable	804 (7.09)	441 (4.99)	363 (14.57)	
Dental visits during the past year				
No	7165 (63.20)	5395 (60.99)	1770 (71.06)	<0.0001
Yes	4172 (36.80)	3451 (39.01)	721 (28.94)	
Self-perceived oral health status				
Good	7033 (62.04)	5704 (64.48)	1329 (53.35)	<0.0001
Poor	4304 (37.96)	3142 (35.52)	1162 (46.65)	
Oral health status				
PT	24.84 ± 0.08	25.51 ± 0.07	22.41 ± 0.19	<0.0001 ^a^
0–9	437 (3.85)	212 (2.40)	225 (9.03)	<0.0001
10–19	940 (8.29)	525 (5.93)	415 (16.66)	
20–28	9960 (87.85)	8109 (91.67)	1851 (74.31)	
Periodontitis				
No	7849 (69.23)	6338 (71.65)	1511 (60.66)	<0.0001
Yes	3488 (30.77)	2508 (28.35)	980 (39.34)	
Relative handgrip strength (kg_BMI_)	1.29 ± 0.01	1.40 ± 0.00	0.88 ± 0.01	<0.0001 ^a^

All values are presented as the mean ± standard error or frequency (n, weighted %). *p*-values were obtained by means of the chi-square test. ^a^ *p*-value was obtained by means of the *t*-test.

**Table 2:** Multivariate regression analysis for PT and relative handgrip strength.

Independent Variables	Crude		Adjusted	
	*β*	*p*-Value	*β*	*p*-Value
PT (continuous)	0.014	<0.0001	0.003	<0.0001
PT (categorical)				
0~9	−0.183	<0.0001	−0.040	0.0056
10~19	−0.179	<0.0001	−0.038	0.0006
20~28	Ref.			

β: regression coefficient. *p*-values were obtained by means of logistic regression analysis. Adjusted for general characteristics (gender, age, educational level, and household income), general health behaviors (smoking, alcohol consumption, exercise, and comorbidi ty), oral health behaviors (frequency of brushing teeth per day, use of oral hygiene products, chewing problem, speaking problem, dental visits during the past year, and self-perceived oral health status), and oral health status (periodontitis).

**Table 3:** Multivariate logistic regression analysis of oral health status and relative handgrip strength.

Independent Variables	Crude	Adjusted
	OR (95% CI)	OR (95% CI)
Periodontitis		
Yes	1.69 (1.51–1.89)	1.02 (0.89–1.16)
No	1	1
PT (continuous)	0.91 (0.90–0.92)	0.97 (0.96–0.99)
PT (categorical)		
0~9	4.33 (3.45–5.44)	1.29 (1.00–1.67)
10~19	3.41 (2.83–4.10)	1.34 (1.10–1.62)
20 ~ 28	1	1

OR: odds ratio, 95% CI: 95% confidence interval. Adjusted for general characteristics (gender, age, educational level, and household income), general health behaviors (smoking, alcohol consumption, exercise, and comorbidity), oral health behaviors (frequency of brushing teeth per day, use of oral hygiene products, chewing problem, speaking problem, dental visits during the past year, and self-perceived oral health status), and oral health status (periodontitis or PT).

## Data Availability

The dataset analyzed for this study can be found at https://knhanes.kdca.go.kr/knhanes/eng/index.do (accessed on 18 November 2021).
